# RIPK3 impacts antibody generation in an induced model of murine lupus through mechanisms other than necroptosis and antigen presentation

**DOI:** 10.3389/fimmu.2025.1506124

**Published:** 2025-09-30

**Authors:** Céleste Pilon, Elena Lonina, Jerrold S. Levine, Sylvie Lesage, Joyce Rauch

**Affiliations:** ^1^ Division of Rheumatology, Department of Medicine, McGill University, Research Institute of the McGill University Health Centre, Montreal, QC, Canada; ^2^ Section of Nephrology, Department of Medicine, University of Illinois at Chicago, Chicago, IL, United States; ^3^ Section of Nephrology, Department of Medicine, Jesse Brown Veterans Affairs Medical Center, Chicago, IL, United States; ^4^ Department of Immunology-Oncology, Maisonneuve-Rosemont Hospital, Montreal, QC, Canada; ^5^ Département de Microbiologie, Infectiologie et Immunologie, Université de Montréal, Montreal, QC, Canada

**Keywords:** systemic lupus erythematosus, receptor-interacting protein kinase 3 (RIPK3), antigen presentation, autoantibodies, murine model, cell death, necroptosis

## Abstract

**Introduction:**

Receptor-interacting protein kinase 3 (RIPK3) is a protein involved in cell death and inflammatory processes. The most recognized function of RIPK3 is the induction of necroptosis, an inflammatory type of cell death that is dependent on RIPK3 kinase activity. Deficiency in RIPK3-dependent pathways has been associated with protection from various inflammatory and autoimmune conditions. Systemic lupus erythematosus (SLE) is an autoimmune disease characterized by the generation of autoantibodies to multiple intracellular antigens leading to multi-organ pathology. Little is known about the involvement of RIPK3-dependent pathways in SLE. We have previously shown that autoantibody generation in an induced model of murine lupus is impaired in RIPK3-deficient mice. The current study aimed to identify the RIPK3-dependent mechanisms that contribute to autoantibody generation in this induced model of murine lupus.

**Methods:**

SLE was induced in C57BL/6 (wild type), RIPK3^-/-^, RIPK^3K51A/K51A^, and MLKL^-/-^ mice by subcutaneous immunization with a mixture of β2-glycoprotein I and lipopolysaccharide in order to evaluate the contribution of RIPK3 and MLKL to autoantibody production in this model. Bone marrow chimeras were generated to investigate the impact of RIPK3 deficiency within the hematopoietic compartment. Antigen presentation assays assessed the impact of RIPK3 deficiency in antigen presenting cells on T cell activation *in vitro*. T cells were evaluated *ex vivo* by flow cytometry following the induction of SLE in wild type and RIPK3-dependent pathway-deficient mice.

**Results:**

Generation of autoantibodies to SLE antigens following immunization with β2-glycoprotein I and lipopolysaccharide was found to be dependent on RIPK3 activity, but independent of MLKL (i.e., RIPK3-dependent necroptosis). Bone marrow chimeric experiments revealed that RIPK3 mediates autoantibody generation through both immune and non-immune compartments. RIPK3 deficiency within antigen presenting cells did not impact T cell activation in vitro. Moreover, early and late T cell activation ex vivo was not impaired in RIPK3-deficient mice following induction of murine lupus.

**Conclusion:**

These results suggest that RIPK3 contributes to autoantibody generation in our induced model of murine lupus through an interplay of pathways that appear to be independent of necroptosis and antigen presentation.

## Introduction

Systemic lupus erythematosus (SLE) is a chronic inflammatory autoimmune disorder affecting over one in 1000 individuals, often with life-long suffering and premature death (1, 2). SLE is characterized by the development of autoantibodies (autoAbs) to self-antigens and damage to multiple organs (3, 4). The origins of SLE remain elusive, and treatment relies mainly on a general suppression of the immune and inflammatory responses (3, 5).

The characteristic antigens targeted by autoAbs in SLE are intracellular and nuclear antigens, which are normally sequestered away from the immune system (3, 6, 7). However, these antigens can be found on the surface of apoptotic cells or released in the context of inflammatory cell death, such as necroptosis (8, 9). In contrast to apoptotic cell death, which is anti-inflammatory and immunosuppressive, necroptotic cell death results in the release of various inflammatory molecules that can act as danger-associated molecular patterns and activate surrounding immune cells and tissues (9–12). The extracellular availability of self-antigens, in combination with an inflammatory environment, may promote the presentation of self-antigens by activated antigen presenting cells (APCs) and the initiation of autoimmune adaptive responses.

Necroptosis can be induced by activation of various cell surface receptors, including Fas, tumor-necrosis-factor (TNF) receptor 1 (TNFR1), and toll-like receptor 4 (TLR4) (9, 13, 14). Following receptor engagement, the induction of necroptosis requires the phosphorylation of receptor interacting protein kinase 3 (RIPK3) (14). Activated RIPK3 then phosphorylates mixed lineage kinase domain-like pseudokinase (MLKL) through its kinase activity, enabling the oligomerization of MLKL and its migration to the cellular membrane to induce necroptosis (14). In addition to its crucial role in initiating necroptosis, RIPK3 can also promote apoptotic cell death in a kinase-independent manner (11, 12, 15).

RIPK3 has garnered a lot of attention in the cancer field for its involvement in cell death pathways. In the case of inflammatory and autoimmune conditions, however, accumulating evidence suggests that the involvement of RIPK3 occurs through cell death-independent mechanisms (11, 12, 15, 16). Such mechanisms include the production of interferon (IFN)-β and NF-κB-dependent pro-inflammatory cytokines (TNF-α, interleukin [IL]-6) by innate immune APCs, both of which require intact RIPK3 kinase activity (11, 12, 17, 18). Signaling through RIPK3 can also result in activation of the inflammasome, and subsequent production of the pro-inflammatory cytokines IL-1β and IL-18 (11, 12). The combined roles of RIPK3 in inflammatory cytokine production and in inflammatory cell death such as necroptosis may synergize to promote RIPK3-mediated inflammatory injury and disease (11, 12). Such a contribution of RIPK3 has been demonstrated in various murine models of autoimmune and inflammatory conditions, including dextran sodium sulfate-induced colitis, psoriatic skin inflammation, autoimmune hepatitis, and rheumatoid arthritis (19–22). However, the contribution of RIPK3 to the development of SLE remains unclear, with protection from disease being strongly dependent on the particular model used for investigation (23–25).

Our laboratory has developed an induced model of murine lupus, in which immunization of nonautoimmune mice with β2-glycoprotein I (β2GPI), a self-antigen targeted in many patients with SLE, and lipopolysaccharide (LPS), an innate immune agonist of TLR4, results in the production of autoAbs targeting multiple SLE autoantigens (27). Antibodies reactive with the immunizing antigen β2GPI (i.e., anti-β2GPI and anti-cardiolipin (CL)) are produced initially, followed by epitope spread to SLE-associated “hallmark” antigens (i.e., double-stranded deoxyribonucleic acid [dsDNA], Ro [SSA], La [SSB], Sm, and Sm/RNP). This model bears striking similarities to human SLE. First, the sequential emergence and specificities of autoantibodies both mimic that seen in human SLE. Moreover, the generation of autoAbs in this model is associated with the development of a β2GPI-specific CD4^+^ T cell response (28). The β2GPI-specific CD4+ T cells share epitopes with β2GPI-reactive T cells from both human autoimmune individuals and mutant mice with spontaneous SLE (28). We previously established that RIPK3 deficiency dampens autoAb production in this model (25). However, the mechanism(s) through which RIPK3 contributes to the initiation of autoimmunity remain(s) to be elucidated.

We demonstrate here that generation of autoAb to SLE self-antigens in our induced model of murine lupus is dependent on RIPK3 activity, but independent of MLKL activity. RIPK3 deficiency within cells of the hematopoietic compartment dampened the generation of β2GPI-specific antibodies, but did not significantly impact the generation of SLE-specific autoAbs. Moreover, RIPK3 deficiency in APCs did not impact T cell activation and IFN-γ production *in vitro*, nor early and late T cell responses *ex vivo*. Together these findings suggest that RIPK3 contributes to the development of murine lupus in our model through a complex interplay of various immune cell-dependent and -independent functions, which are independent of necroptosis and do not impact antigen presentation.

## Materials and methods

### Mice

Specific pathogen-free female C57BL/6 wild type (WT) mice (12–16 weeks of age) were bred in-house using breeders from The Jackson Laboratory (Bar Harbor, ME). RIPK3^-/-^, which originated from Vishva Dixit (Genentech, San Francisco, CA) (29) were generously provided by Dr. Maziar Divangahi (McGill University) (18). RIPK3^K51A/K51A^ mice initially described by Mandal et al. (30) were kindly supplied by GlaxoSmithKline Inc. (GSK; Collegeville, PA). MLKL^-/-^ was generated by Dr Jiahuai Han (Xiamen University (31) and breeders were generously provided by Dr. Maya Saleh (McGill University). All deficient strains were on a C57BL/6 background and bred in-house. Mice were maintained and bred according to Canadian Council on Animal Care (CCAC) guidelines (consistent with the National Research Council Guide for the Care and Use of Laboratory Animals [8th Edition, 2011]), and maintained on food and water *ad libitum*. Animal experiments were approved by the McGill University Animal Care Committee.

### Reagents

Unless stated otherwise, all reagents were obtained commercially from the following sources and used without further purification: human β2-glycoprotein I (β2GPI) (Crystal Chem, Downers Grove, IL); and bovine heart cardiolipin (CL) (Avanti Polar Lipids, Elk Grove Village, IL). Lipopolysaccharide (LPS; *Escherichia coli*-derived, serotype O111:B4) was obtained from List Biological Laboratories, Campbell, CA or Sigma-Aldrich Canada Co., Oakville, ON. Other reagents are indicated under the protocol in which they were used.

### Induction of a model of murine lupus

Mice (12–16 weeks) were immunized subcutaneousy with a mixture of β2GPI (20μg) and LPS (10μg) every 14 days for a total of 3 immunizations, unless otherwise indicated. This immunization protocol with β2GPI and LPS results in the spread of the immune response to IgG autoAbs typically seen in human SLE (including to Ro[SSA], La[SSB], dsDNA, nRNP, and Sm), compared immunization with buffer or LPS alone (27). Mice immunized with LPS alone were included as a control group in each experiment. Serum was collected from saphenous vein bleeds 10 days following each immunization to assess serum autoAb levels.

### Detection of autoAbs to β2GPI, cardiolipin, and hallmark SLE antigens

Development of SLE was assessed by performing in-house enzyme-linked immunosorbent assays (ELISAs) to detect serum autoAbs against β2GPI, CL, and hallmark SLE antigens (dsDNA, Ro [SSA], La [SSB], Sm, and Sm/RNP), as previously described (26, 27).

### Characterization of immune cell populations at baseline

The spleen, lymph nodes (LN; axial, brachial and inguinal), and thymus were harvested from WT, RIPK3^-/-^, RIPK3^K51A/K51A^ and MLKL^-/-^ mice. The spleens and LN were treated with collagenase V (1mg/mL; MilliporeSigma) for 15 minutes at 37^°^C and filtered through a 70 μm nylon cell strainer to obtain single cell suspensions. Splenocytes were depleted of red blood cells (RBCs) by washing with ammonium-chloride-potassium (ACK) lysis buffer. 1x10^6^ cells/well were stained with fluorescently conjugated antibodies for surface expression of lineage-specific markers and acquired by flow cytometry on a BD LSRFortessa™ Cell Analyzer (BD Biosciences).

### T cell stimulation assay

Spleens were harvested from WT, RIPK3^-/-^, RIPK3^K51A/K51A^, and MLKL^-/-^ mice under sterile conditions and processed into single cell suspensions. T cells were isolated from the splenocyte suspensions by negative selection (Stemcell) and stained with CellTrace Violet™ (CTV). 5x10^5^cells/well were plated in 24-well plates coated with anti-CD3 (clone 145-2C11) and anti-CD28 (clone 37.51), and incubated for 72 h at 37°C (5% CO_2_). Cells were harvested and stained for surface expression of CD4 and CD8 and acquired by flow cytometry on a BD LSRFortessa™ Cell Analyzer (BD Biosciences). IFN-γ concentration in the supernatant was measured by ELISA, according to the manufacturer’s instructions (BD Biosciences).

### Bone marrow chimeras of WT and RIPK3^-/-^ mice

C57BL/6 WT mice expressing the congenic marker CD45.1 were treated with bacitracin in drinking water and irradiated with a dose of 9.5Gy. Bone marrow cells were isolated from the femurs of WT and RIPK3^-/-^ CD45.2^+^ donor mice, and T cells were depleted using negative selection. Within 24h of irradiation, 4x10^6^ bone marrow cells from either WT or RIPK3^-/-^ mice were transferred intravenously to WT CD45.1^+^ mice. Immune reconstitution was assessed on peripheral blood at 11 weeks following bone marrow transfer. The murine lupus model was induced in the bone marrow chimeras 12 weeks post-transfer.

### Generation and stimulation of BMDCs

Bone marrow cell suspensions were isolated from the femurs of mice and RBCs were depleted using ACK lysis buffer. Bone marrow-derived dendritic cells (BMDCs) were generated from bone marrow cells following a 7-day culture in the presence of GM-CSF (20 ng/mL; PeproTech). BMDCs were plated at 1x10^6^cells/well in 12-well plates and stimulated with LPS (10 ng/mL) for 16 h. Cells were harvested by scraping, stained for MHC II and co-stimulatory molecule expression (CD80, CD86), and acquired by flow cytometry on a BD LSRFortessa™ Cell Analyzer (BD Biosciences). BMDCs were defined as CD11c^+^ MHC-II^hi^ cells. IL-6 and TNFα in the supernatants were measured by ELISA (BD Biosciences).

### Antigen presentation assays

#### Antigen presentation by CD4-depleted splenocytes

Spleens were harvested from WT, RIPK3^-/-^, RIPK3^K51A/K51A^, MLKL^-/-^, and OT-II mice, processed into a single cell suspension, and depleted of RBCs using ACK lysis buffer. The cell suspension was passed through a 70 µm filter, incubated with CD4 microbeads (L3T4, Miltenyi Biotec) and CD4^+^ and CD4^-^ (CD4-depleted splenocytes) fractions were isolated using autoMACS (Miltenyi Biotec). The CD4-depleted splenocytes from WT, RIPK3^-/-^, RIPK3^K51A/K51A^ and MLKL^-/-^ mice were treated with mitomycin C (20 μg/mL) for 2 h, washed and plated at 200,000 cells/well in 96-well plates. The CD4^+^ T cell fraction from OT-II mice was stained with CellTrace Violet™ (CTV) and 50,000 cells/well were added to the mitomycin C-treated, CD4-depleted splenocytes. Ovalbumin (OVA)_323-339_ (10µg) or human serum albumin (HSA, control) was added to the co-culture, and incubated for 72h at 37^°^C (5% CO_2_).

#### Antigen presentation by BMDCs

BMDCs were plated at 2.5x10^5^ cells/well in 48-well plates and stimulated with LPS (10 ng/mL) for 2 h at 37^°^C (5% CO_2_). BMDCs were then incubated with ovalbumin (OVA; 10 μg/mL) or decreasing concentrations of the OVA_323–339_ peptide (5 μg/mL, 5-fold dilutions) for 16 h at 37^°^C (5% CO_2_). The next day, T cells were isolated from the spleen of OT-II mice by negative selection (Stemcell), stained with CTV and co-cultured with antigen-loaded BMDCs for 72 h at 37^°^C (5% CO_2_).

#### Harvesting and staining

Phorbol 12-myristate 13-acetate (PMA) (20 ng/mL), ionomycin (750 ng/mL), and monensin (1x) were added to the wells during the last 3 hours of incubation. Cells were harvested by pipetting and stained for viability, CD4, CD8, and IFN-γ. Cells were acquired by flow cytometry on a BD LSRFortessa™ Cell Analyzer (BD Biosciences).

#### Characterization of dendritic cell activation and early T cell responses in lymph nodes

The draining (inguinal) and non-draining (pooled brachial and axillary) LNs were harvested from WT, RIPK3^-/-^, RIPK3^K51A/K51A^, and MLKL^-/-^ mice 2 days after the 4^th^ immunization with β2GPI and LPS or LPS only. The LNs were crushed through a 70 μm nylon cell strainer using a syringe plunger to obtain single cell suspensions. Cell suspensions were depleted of RBCs by washing with ACK lysis buffer. 1x10^6^ cells were added to 96-well plates, stained with fluorochrome-conjugated antibodies for surface expression of lineage-specific and activation markers, and acquired by flow cytometry on a BD LSRFortessa™ Cell Analyzer (BD Biosciences).

#### Stimulation and characterization of co-stimulatory markers on B cells

Spleens were harvested from WT, RIPK3^-/-^, RIPK3^K51A/K51A^, and MLKL^-/-^ mice under sterile conditions and processed into a single cell suspension. B cells were isolated from the splenocyte suspension by negative selection (Stemcell), and 1x10^6^cells/well were plated in 24-well plates. B cells were stimulated with LPS (10 ng/mL) for 16h at 37^°^C (5% CO_2_) or left unstimulated. Cells were harvested and stained for surface expression co-stimulatory markers (MHC II, CD80 and CD86) and acquired by flow cytometry on a BD LSRFortessa™ Cell Analyzer (BD Biosciences).

#### Characterization of the T and B cell responses in lymph nodes

WT, RIPK3^-/-^, RIPK3^K51A/K51A^, and MLKL^-/-^ mice were immunized subcutaneously with β2GPI and LPS, LPS only, or PBS, every 14 days for a total of 3 immunizations. Draining (inguinal) and non-draining (pooled brachial and axillary) LNs were collected 8 days following the third immunization, homogenized, and counted. 1x10^6^ cells were stained for expression of T and B cell markers and acquired by flow cytometry.

#### ELISpot of IgG and β2GPI-specific IgG antibody-producing cells

WT, RIPK3^-/-^, RIPK3^K51A/K51A^, and MLKL^-/-^ mice were immunized subcutaneously with β2GPI and LPS for a total of 3 immunizations. Draining (inguinal) LNs were collected 8 days following the 3^rd^ immunization, homogenized, and counted. ELISpot was performed using a Mouse IgG Single-Color ELISPOT 96-well white kit (Cellular Technologies Limited, Shaker Heights, OH) according to the manufacturer’s protocol for the detection and quantification of whole IgG- and β2GPI-specific IgG-producing cells.

## Results

### RIPK3 deficiency dampens autoantibody production following immunization with β2GPI and LPS independently of necroptosis

**Figure 1:**
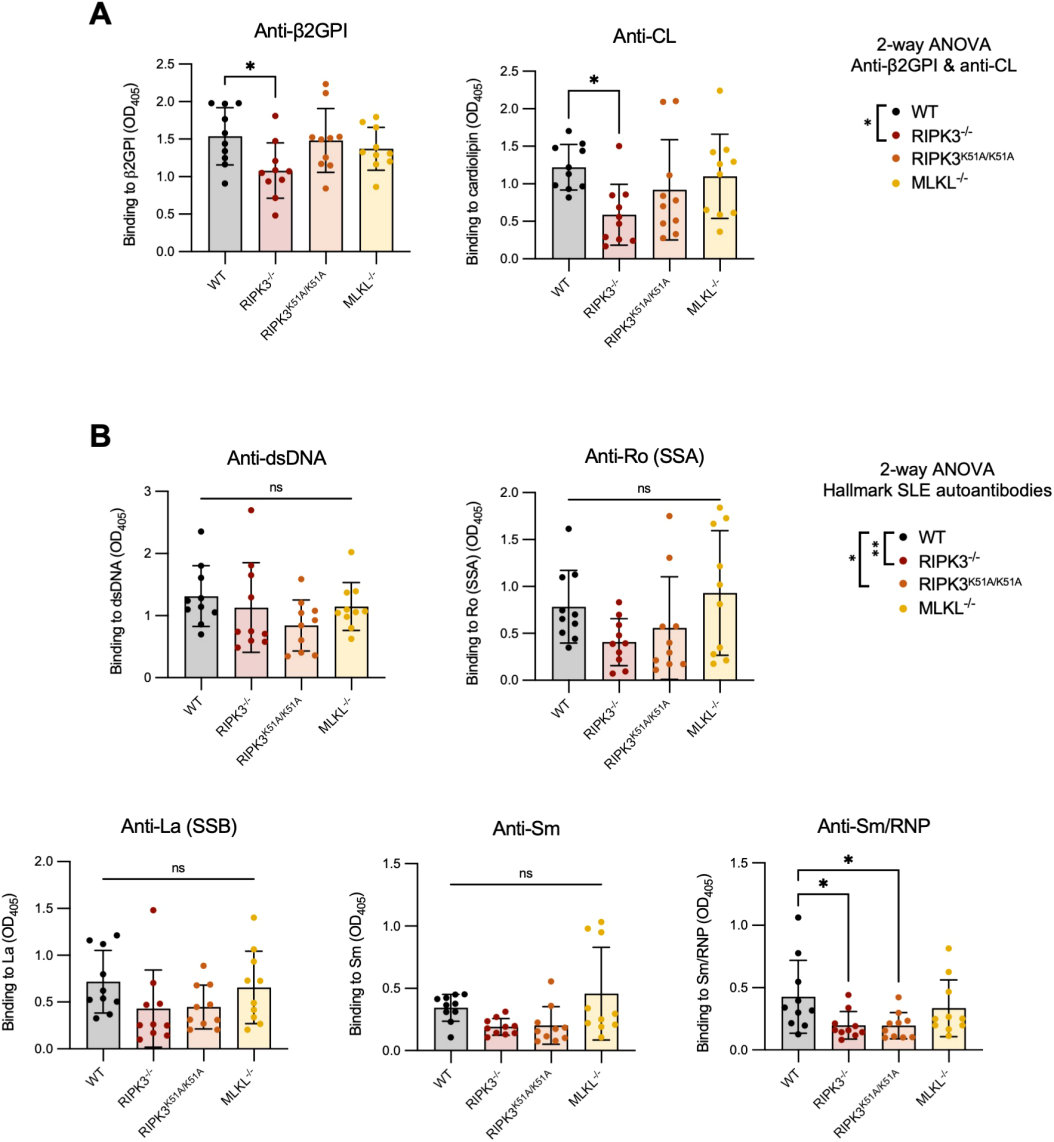
RIPK3^-/-^ and RIPK3^K51A/K51A^ mice have decreased levels of autoAbs following immunization with β2GPI+LPS. C57BL/6 WT, RIPK3^-/-^, RIPK3^K51A/K51A^ and MLKL^-/-^ mice received 3 subcutaneous immunizations with β2GPI (20ug) and LPS (10ug), followed by an intravenous injection of LPS (0.5μg) 24h later. **(A, B)** Serum autoAbs to **(A)** β2GPI and cardiolipin (CL) and **(B)** hallmark SLE autoantigens (dsDNA, Ro[SSA], La[SSB], Sm, Sm/RNP) were determined by ELISA, where the mean autoAb levels (OD_405_) of mice immunized with LPS only (without β2GPI) (n=6) was ≤0.3. Data were pooled from two independent experiments. In all graphs, error bars represent mean ± SD; each data point represents an individual mouse. Individual autoAbs were analyzed by one-way ANOVA followed by a Dunnett’s *post-hoc* test to compare each group to the WT control group. Additionally, autoAbs were analyzed as combined subsets: **(A)** anti-β2GPI and anti-CL antibodies and **(B)** “hallmark autoAbs” (anti-dsDNA, Ro[SSA], La[SSB], Sm, and Sm/RNP) by two-way ANOVA followed by a Tukey’s multiple comparisons *post-hoc* test. This analysis was included as an inserted legend to the right of the graphs for each subset. *P<0.05 and **P<0.01; ns, nonsignificant.

RIPK3-dependent cell death and inflammation occurs through both kinase-dependent and -independent (scaffolding) functions. To investigate which of these mechanisms are involved in the generation of SLE-associated autoAbs, we induced our model of murine lupus in wild-type (WT), RIPK3-deficient (RIPK3^-/-^), and RIPK3 kinase-dead (RIPK3^K51A/K51A^) mice. MLKL-deficient (MLKL^-/-^) mice were also included to investigate the contribution of RIPK3-dependent necroptosis. RIPK3^-/-^ mice developed significantly lower levels of autoAbs to the immunizing antigen β2GPI and the closely related antigen CL, compared to WT mice (**Figure 1A**). This was also the case when anti-β2GPI and anti-CL antibodies were analyzed as a combined antibody subset (2-way ANOVA) (**Figure 1A**). RIPK3^-/-^ mice also had significantly reduced levels of autoAbs to hallmark SLE antigens when the antibodies were compared as a combined subset, but only anti-Sm/RNP antibodies were significantly reduced when individual autoAbs were compared to those of WT mice (**Figure 1B**). Like RIPK3^-/-^ mice, RIPK3^K51A/K51A^ mice had reduced levels of the combined subset of hallmark SLE autoAbs, but only reduced levels of anti-Sm/RNP when individual autoAbs were compared to those of WT mice. The levels of anti-β2GPI and anti-CL autoAb in RIPK3^K51A/K51A^ mice were comparable to those of WT mice (**Figures 1A, B**). In contrast to the RIPK3-deficient strains, MLKL^-/-^ did not differ from WT mice in their production of SLE antibodies. MLKL deficiency did not impact autoAb production, suggesting that autoAb production in our murine lupus model results from necroptosis-independent mechanisms of RIPK3 (**Figures 1A, B**).

Deficient strains were compared to C57BL/6 WT mice, their background strain. However, we compared C57BL/6 WT mice to RIPK3^K51A/K51A^ WT littermates (RIPK3^WT/WT^) to ensure that their antibody response was similar. Both strains showed equivalent anti-β2GPI, anti-CL, and anti-dsDNA antibody levels after 2 immunizations with β2GPI and LPS (**Supplementary Figure 1**). A shortened immunization protocol was used in this experiment as the purpose was solely the comparison of autoAb induction between strains. We conclude that the response of RIPK3^WT/WT^ mice is comparable to that of C57BL/6 WT mice for induction of the murine lupus model, and that C57BL/6 WT mice are therefore a suitable control in this context.

### RIPK3 deficiency dampens autoantibody production following immunization with β2GPI and LPS through effects on both the immune and non-immune compartments

To confirm that the impact of RIPK3 on autoAb production was not due to differences in immune cell composition among strains, we characterized the immune cell proportions in WT, RIPK3^-/-^, RIPK3^K51A/K51A^, and MLKL^-/-^ mice prior to induction of the murine lupus model. We focused on cells involved in antigen presentation and antibody production. No significant differences were observed in the cell numbers or proportions of conventional dendritic cells (cDCs), CD4^+^ T cells, or B cells in the lymph nodes (LNs) and spleens among WT, RIPK3^-/-^, RIPK3^K51A/K51A^, and MLKL^-/-^ mice (**Supplementary Figure 2**). A complete summary of the immune cell proportions in the LNs, spleen, and thymus is included in **Supplementary Table 1**.

**Figure 2:**
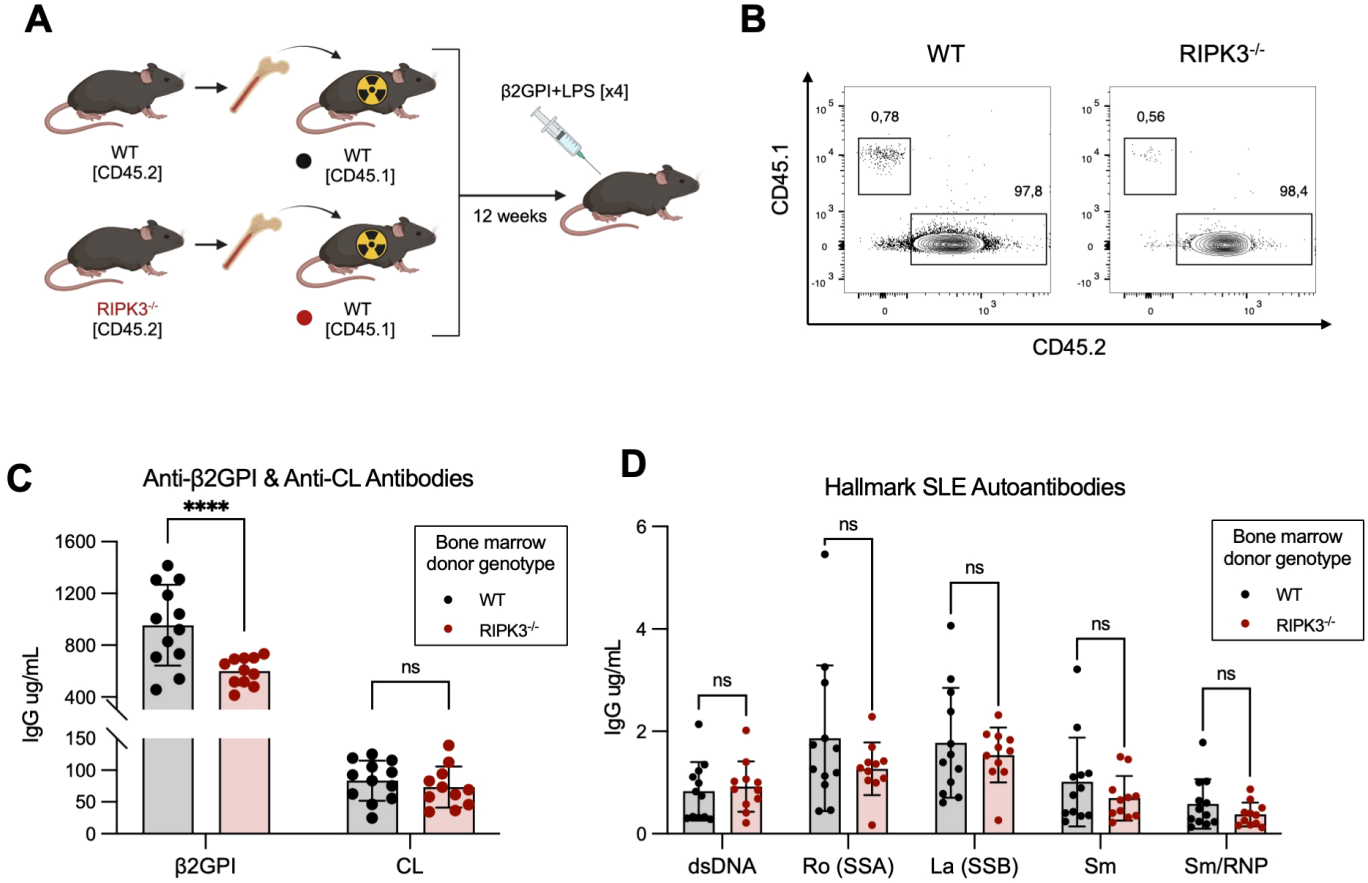
RIPK3 deficiency in the immune compartment dampens autoAb generation following immunization with β2GPI+LPS. **(A)** Bone marrow cells isolated from CD45.2^+^ WT and RIPK3^-/-^ mice were transferred to irradiated CD45.1^+^ WT mice. 12 weeks following bone marrow cell transfer, mice were immunized as per induction of our model of murine lupus, for a total of 4 immunizations. Schematic created with BioRender.com. **(B)** Proportions of CD45.1^+^ and CD45.2^+^ cells in the peripheral blood, 11 weeks following bone marrow transfer. **(C, D)** Serum autoAb titers were assayed by ELISA and quantified using an IgG standard curve. Comparisons for autoAbs between WT and RIPK3^-/-^ mice were analyzed using a two-way ANOVA followed by a Tukey’s multiple comparisons *post-hoc* test. Data were generated in a single experiment. In all graphs, error bars represent mean ± SD; each data point represents an individual mouse. ****P<0.0001; ns, nonsignificant.

To investigate whether RIPK3 reduced autoAb generation in our model of murine lupus through an effect on immune cell function, we created bone marrow chimeras in which the hematopoietic cells of WT mice were replaced by either WT or RIPK3^-/-^ immune cells (**Figures 2A, B**). Mice received 4 immunizations to mitigate any potentially immunosuppressive effect that generation of the chimeric state may have had on the immune response to injected antigens. RIPK3 deficiency in the hematopoietic cell compartment resulted in significantly lower levels of autoAbs to the immunizing antigen (β2GPI), but not to CL, compared to WT mice (**Figure 2C**). However, no differences in the levels of autoAbs to hallmark SLE autoantigens were observed between WT and RIPK3^-/-^ bone marrow chimera mice (**Figure 2D**).

These results suggest that RIPK3 signaling in immune cells may be dispensable for epitope spread to hallmark SLE autoantibodies in our model, but is important for generating antibodies to the immunizing antigen β2GPI. Since we know that autoAb production in our model is associated with the generation of a β2GPI-specific CD4^+^ T cell response (28), we next focused our investigations on immune cell-dependent mechanisms through which RIPK3 may influence the generation of antibodies to β2GPI.

We first confirmed the importance of a T cell response in the generation of anti-β2GPI antibodies in our model. Two days following the 4^th^ immunization with β2GPI and LPS, we observed a specific expansion of T follicular helper (T_FH_) cells in the draining LN compared to PBS-immunized mice (**Supplementary Figure 3**). T_FH_ cells play a crucial role in the establishment of a germinal center (GC) where T cell-dependent activation of B cells takes place (32, 33). Accordingly, we found an expansion of GC B cells and IgG^+^ class-switched B cells in the draining LN 8 days following the 3^rd^ immunization with β2GPI and LPS (**Supplementary Figure 4**). These findings suggest that B cell maturation and autoAb production in our model of murine lupus is driven by a classical T cell-dependent GC reaction (33). Together, these results reinforce the importance of the T cell response for autoAb production in our model, and further motivate a focus on the role of RIPK3 deficiency in the T cell-dependent generation of β2GPI-specific antibodies.

### RIPK3 deficiency does not impact the expression of antigen presentation markers by DCs

The establishment of a robust T cell response requires the complex integration of three major signals provided by the APC: [1] antigen processing and peptide presentation on the major histocompatibility complex class II (MHC II), [2] co-stimulatory molecule expression by the APC, and [3] specific cytokine production by the APC (32, 34, 35).

**Figure 3:**
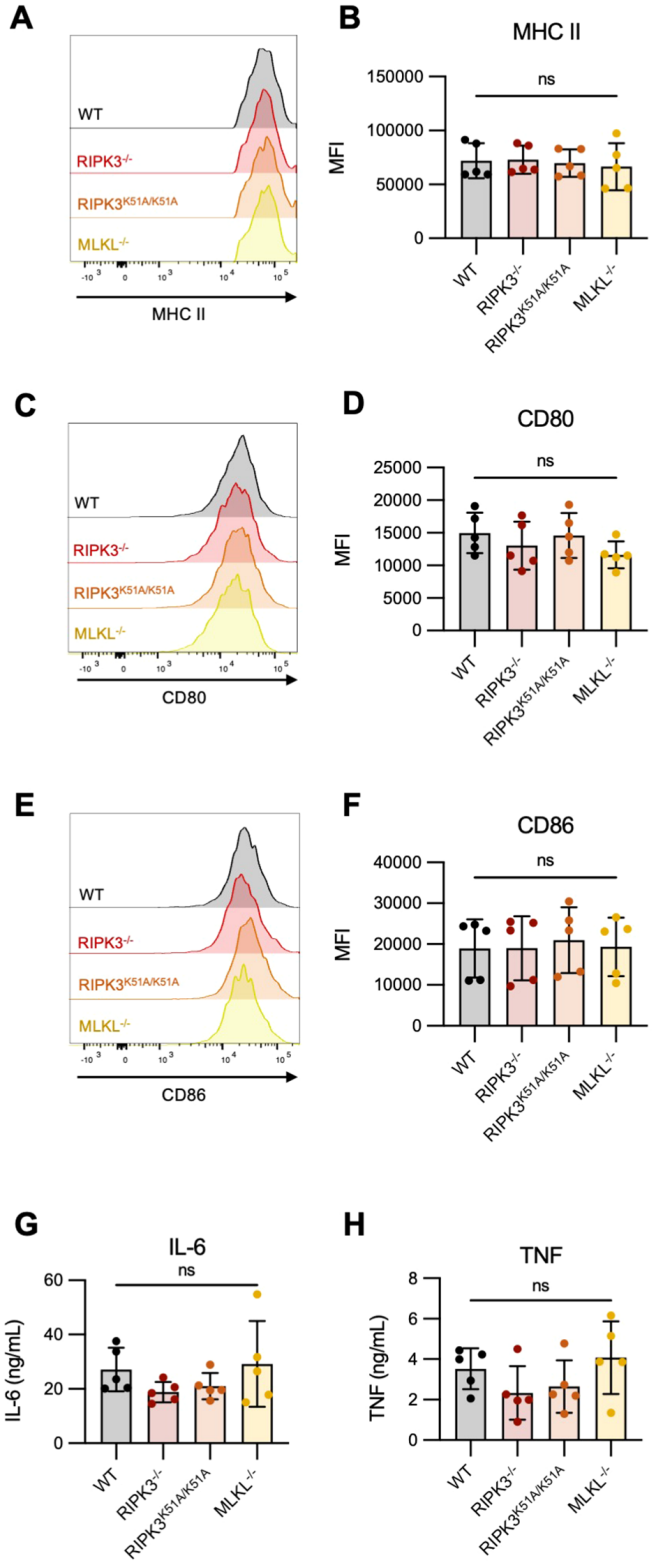
Expression of antigen presentation markers and pro-inflammatory cytokine production is comparable between RIPK3-deficient and WT BMDCs. Bone marrow-derived dendritic cells (BMDCs) were generated from the bone marrow of WT, RIPK3^-/-^, RIPK3^K51A/K51A^, and MLKL^-/-^ mice. BMDCs were stimulated with LPS (10 ng/mL) for 16h. **(A-F)** Surface expression of MHC-II, CD80 and CD86 was quantified by flow cytometry. **(A, B)** Representative histograms **(A)**, and mean fluorescence intensity (MFI) **(B)** of MHC-II expression on LPS-stimulated BMDCs. **(C, D)** Representative histograms **(C)**, and MFI **(D)** of CD80 expression on LPS-stimulated BMDCs. **(E, F)** Representative histograms **(E)**, and MFI **(F)** of CD86 expression on LPS-stimulated BMDCs. **(G-I)** IL-6 **(G)** and TNF-α **(H)** concentrations were quantified in the cell culture supernatant of LPS-stimulated BMDCs by ELISA. Data were pooled from five independent experiments. In all graphs error bars represent mean ± SD; each data point represents one independent experiment. Data were analyzed using a one -way ANOVA. ns, nonsignificant.

We first investigated if deficiency in RIPK3-dependent pathways impacted the expression of MHC II (signal 1) and/or co-stimulatory molecules (signal 2) by bone marrow-derived DCs (BMDCs), following their activation with LPS. We found that expression of MHC-II, CD80, and CD86 was comparable between WT and RIPK3-dependent pathway-deficient BMDCs following LPS stimulation (**Figures 3A–F**). We next determined if the production of pro-inflammatory cytokines (signal 3) was impaired in RIPK3-deficient BMDCs. We found a slight but non-significant decrease in the production of TNF-α and IL-6 by RIPK3^-/-^ and RIPK3^K51A/K51A^ BMDCs compared to WT and MLKL^-/-^ BMDCs (**Figures 3G, H**). These data indicate that RIPK3 deficiency does not significantly impact any of the three major APC signals necessary for T cell activation.

### RIPK3 deficiency in APCs does not impact the phenotype or activation of T cells following antigen presentation *in vitro*

To determine whether RIPK3-deficiency in APCs impacts T cell activation (following antigen presentation), we performed an *in vitro* antigen presentation assay using antigen-specific OT-II T cells that are activated by presentation of the ovalbumin (OVA) peptide 323-339 (OVA_323-339_) on MHC II. In these experiments, we investigated multiple aspects of the overall T cell response.

**Figure 4:**
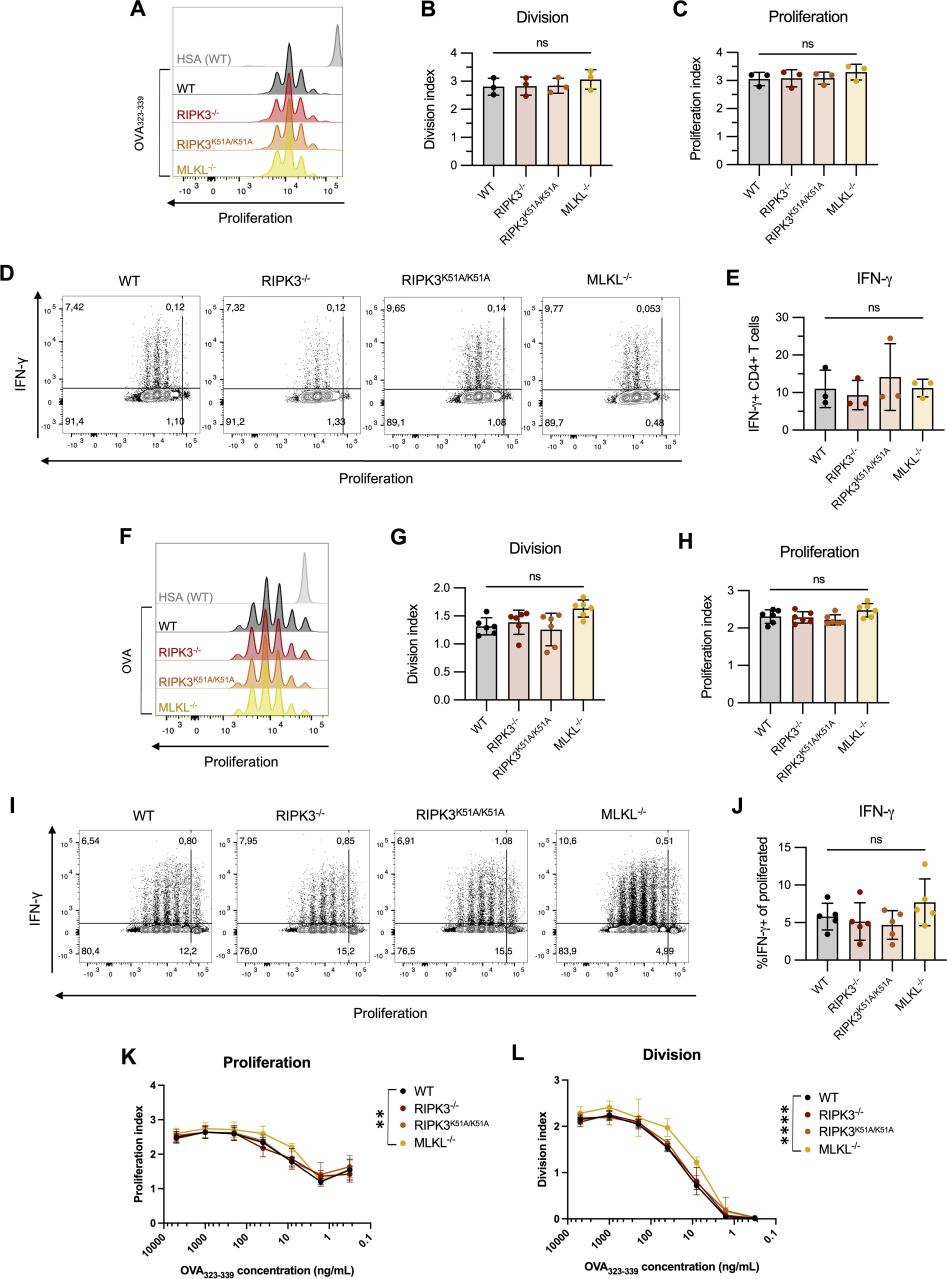
RIPK3 deficiency in BMDCs does not impact T cell proliferation following antigen presentation *in vitro*. **(A-E)** CTV-stained OT-II CD4^+^ T cells were incubated with the ovalbumin (OVA)_323–339_ peptide (10µg/mL) in the presence of CD4-depleted splenocytes isolated from WT, RIPK3^-/-^, RIPK3^K51A/K51A^, or MLKL^-/-^ mice for 72h. Proliferation and IFN-γ production were detected by flow cytometry. Incubation with human serum albumin (HSA) was used as a control (grey). **(A-C)** Representative histogram **(A)**, division **(B)** and proliferation index **(C)** of OT-II CD4^+^ T cells. **(D, E)** Representative flow plots **(D)** and proportion of IFN-γ producing CD4^+^ T cells **(E)**. Data were pooled from three independent experiments. Data were analyzed using a one-way ANOVA, followed by a Dunnett’s *post-hoc* test to compare each group to the WT control group. **(F-J)** CTV-stained OT-II CD4^+^ T cells were incubated with OVA-loaded BMDCs derived from WT, RIPK3^-/-^, RIPK3^K51A/K51A^, or MLKL^-/-^ mice for 72h. **(F-H)** Representative histogram **(F)**, proliferation **(G)** and division index **(H)** of OT-II CD4^+^ T cells. **(I, J)** Representative flow plots **(I)** and proportion of IFN-γ producing CD4^+^ T cells **(J)**. Data were pooled from six **(F-H)** or five **(I, J)** independent experiments. Data were analyzed using a one-way ANOVA, followed by a Dunnett’s *post-hoc* test to compare each group to the WT control group. **(K-L)** OT-II CD4^+^ T cells were incubated for 72h with BMDCs derived from WT, RIPK3^-/-^, RIPK3^K51A/K51A^, or MLKL^-/-^ mice loaded with decreasing concentrations of OVA_323-339_. Proliferation **(K)** and division **(L)** index of OT-II CD4^+^ T cells. Data were pooled from five independent experiments. Data were analyzed using a one-way ANOVA, followed by a Dunnett’s *post-hoc* test to compare each group to the WT control group. In all graphs, error bars represent mean ± SD; each data point represents one independent experiment. **P<0.01 and ****P<0.0001; ns, nonsignificant.

First, we found that a deficiency in RIPK3-dependent pathway in CD4-depleted splenocytes did not impact the proliferation of OT-II T cells following antigen-specific activation (**Figures 4A–C**). Next, we examined cytokine production by activated OT-II T cells. We have previously shown that development of murine lupus in our model is associated with the emergence of IFN-γ-producing β2GPI-specific T cells (28). Similar to proliferation, IFN-γ production by OT-II T cells was comparable following antigen presentation by either WT or RIPK3-dependent pathway-deficient splenocytes (**Figures 4D, E**).

To this point, we had only used the processed peptide, OVA_323-339_, in our antigen presentation assays. To determine whether RIPK3 might impact antigen presentation by altering intracellular antigen processing, we co-incubated WT and RIPK3-dependent pathway-deficient LPS-stimulated BMDCs with intact OVA protein, and then used these BMDCs to assess the impact of RIPK3 deficiency on T cell activation and IFN-γ production. As seen with processed OVA peptide, antigen presentation with the additional requirement for processing of the intact protein was unaffected by RIPK3 deficiency. T cell proliferation (**Figures 4F–H**) and IFN-γ production (**Figures 4 I, J**) by OT-II T cells were comparable whether they were co-incubated with WT or RIPK3-dependent pathway-deficient BMDCs.

Finally, we explored the possibility that RIPK3 deficiency may affect the concentration of antigen required for effective antigen presentation. OT-II T cells were co-incubated with LPS-activated BMDCs from WT or RIPK3-dependent pathway-deficient mice in the presence of decreasing concentrations of OVA_323-339_. The dose-response curves for proliferation of OT-II T cells were virtually indistinguishable following antigen presentation by RIPK3^-/-^, RIPK3^K51A/K51A^, or WT BMDCs (**Figures 4K, L**). However, a significant increase in the proliferation of OT-II T cells was observed following co-incubation with MLKL^-/-^ BMDCs compared to WT BMDCs (**Figures 4K, L**). The significance of this finding is uncertain, although it suggests that MLKL deficiency in APCs may impact T cell proliferation through RIPK3-independent mechanisms (**Figures 4K, L**).

Overall, RIPK3 deficiency in APCs did not affect the *in vitro* activation of T cells, as assessed by either proliferation or IFN-γ production. These findings suggest that factors extrinsic to the APC may influence antigen presentation and T cell activation in RIPK3-deficient mice.

### RIPK3 deficiency in APCs does not impact the phenotype or activation of T cells following antigen presentation *ex vivo*

The outcome of activation of RIPK3-dependent pathways is dependent on a combination of inflammatory triggers and the cell-specific environment in which they take place (11, 12). Given the limited complexity of an *in vitro* antigen presentation assay compared to that of T cell activation *in vivo*, we next sought to determine the impact of deficiency in RIPK3-dependent pathways on early T cell activation *ex vivo*.

Deficiency in RIPK3-dependent pathways did not impair the *in vitro* activation or IFN-γ production by freshly isolated splenic T cells (**Supplementary Figure 5**). We therefore conclude that any effects of RIPK3-dependent pathways on T cell activation may be a consequence of extrinsic effects during their activation.

**Figure 5:**
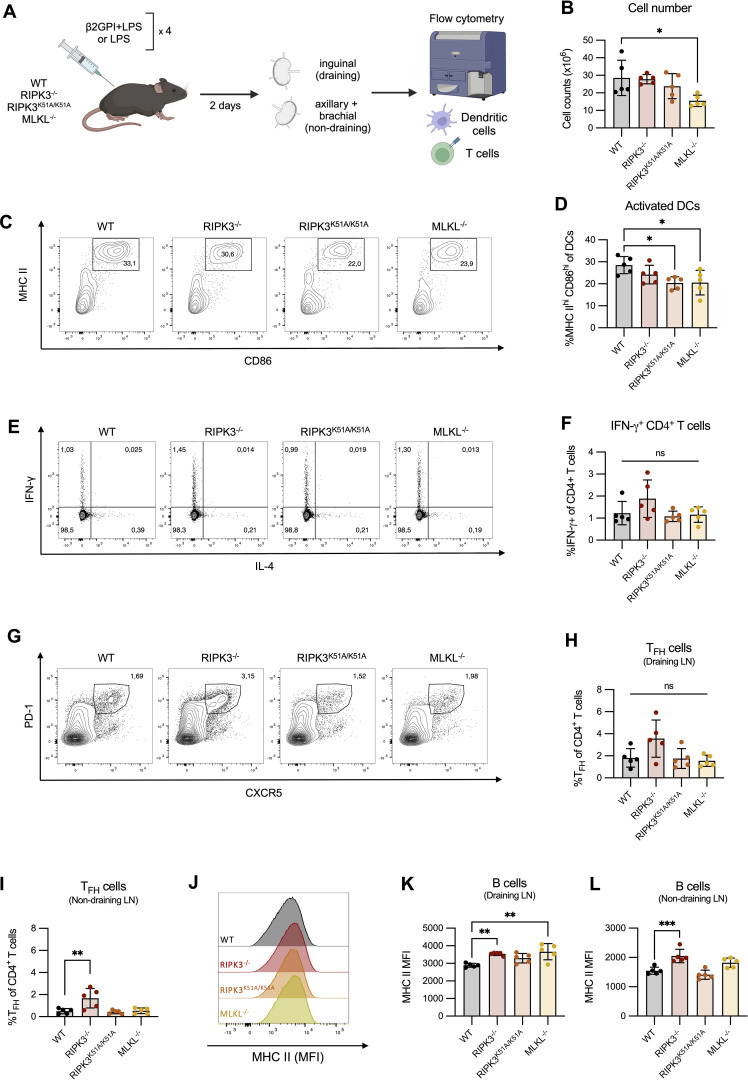
RIPK3 deficiency does not impair the *in vivo* T cell response following immunization with β2GPI and LPS. **(A)** WT, RIPK3^-/-^, RIPK3^K51A/K51A^ and MLKL^-/-^ mice were immunized as per induction of our model of murine lupus, for a total of 4 immunizations. Draining (inguinal) and non-draining (axillary and brachial) lymph nodes (LN) were collected 2 days following the 4^th^ immunization. Cell suspensions were counted and 1x10^6^ cells were stained for T and B cell markers and acquired by flow cytometry. Schematic created with BioRender.com. **(B)** Cell numbers in the draining LN. **(C, D)** Representative flow plots **(C)** and proportions **(D)** of activated (MHC-II^hi^ CD86^hi^) DCs in the draining LN. **(E, F)** Representative flow plots **(E)** and proportions **(F)** of IFN-γ producing CD4^+^ T cells in the draining LN. **(G, H)** Representative flow plots **(G)** and proportions **(H)** of T_FH_ (CXCR5^+^ PD-1^+^) CD4^+^ T cells in the draining LN. **(I)** Proportion of T_FH_ (CXCR5^+^ PD-1^+^) CD4^+^ T cells in the non-draining LN. **(J-L)** Representative histogram **(J)**, and mean fluorescence intensity **(K-L)** of MHC-II expression on B cells in the draining **(J, K)** and non-draining **(L)** LN. Data were generated in a single experiment. In all graphs error bars represent mean ± SD; each data point represents an individual mouse. Data were analyzed using a one-way ANOVA, followed by a Dunnett’s *post-hoc* test to compare each group to the WT control group. *P<0.05, **P<0.01 and ***P<0.001; ns, nonsignificant.

Using our induced model of murine lupus, we evaluated the impact of RIPK3 on APC and T cell activation *ex vivo* (**Figure 5A**). Here, mice received an additional immunization following the induction of our model in order to evaluate the immune response at a timepoint suitable for T cell activation. The total number of cells in the draining LN 48 h after a 4^th^ immunization with β2GPI and LPS was comparable between WT and RIPK3-dependent pathway-deficient mice (**Figure 5B**). However, a decreased proportion of activated DCs (MHC II^hi^ CD86^hi^) was found in the draining LN of RIPK3^K51A/K51A^ and MLKL^-/-^ mice, compared to WT mice (**Figures 5 C, D**). No difference in the proportion of activated DCs was observed between RIPK3^-/-^ and WT mice (**Figures 5 C, D**). A change in the proportion of activated DCs could impact T cell phenotype or proportion in the draining LN. We next investigated the proportion of CD4^+^ IFN-γ-producing T cells, as they have previously been associated with the β2GPI-specific T cell response in our model (28). IL-4 was included to demonstrate the selectivity of cytokine expression towards a Th1 profile. Despite significant differences in the proportion of activated DCs in RIPK3^K51A/K51A^ and MLKL^-/-^ mice as compared to WT mice, no differences in the proportions of IFN-γ-producing CD4^+^ T cells or T_FH_ cells were observed between WT and RIPK3-dependent pathway-deficient mice (**Figure 5E–H**). The slight increase in the proportion of T_FH_ observed in the draining LN of RIPK3^-/-^ mice appeared to be independent of the response to immunization with β2GPI and LPS, as a greater increase in the proportion of T_FH_ cells was observed in the non-draining LN of RIPK3^-/-^ mice, compared to WT mice (**Figures 5G–I**). For Panels C-L in **Figure 5**, absolute cell counts were performed and did not differ from cell proportions (data not shown).

B cells can also act as APCs and indeed were found to upregulate their expression of MHC II following induction of our model of murine lupus (**Supplementary Figures 3E, F**). We therefore evaluated the expression of MHC II by B cells in WT and RIPK3-dependent pathway-deficient mice. Although we observed an increased expression of MHC II by B cells in the draining LN of RIPK3^-/-^ and MLKL^-/-^ mice compared to WT mice (**Figures 5 J, K**), elevated MHC II expression by RIPK3^-/-^ B cells was also observed in the non-draining LN. This finding suggests that RIPK3 deficiency may enhance MHC II expression by B cells independently of induction of our model of murine lupus (**Figure 5L**).

Nonetheless, these *ex vivo* differences in MHC II expression by RIPK3-deficient B cells warranted a closer examination of the impact of RIPK3 deficiency on B cell activation *in vitro*. No significant differences in the expression of MHC II, CD80, or CD86 were identified between unstimulated WT and RIPK3-dependent pathway-deficient splenic B cells (**Supplementary Figures 6A, B**). In contrast, following LPS stimulation, a significant increase in the level of MHC II expression was observed on B cells from RIPK3^-/-^ mice compared to WT mice (**Supplementary Figures 6C, D**). CD80 and CD86 expression between RIPK3^-/-^ and WT B cells was comparable (**Supplementary Figures 6C, D**). All differences were limited to RIPK3^-/-^ B cells. No differences in MHC II or co-stimulatory marker expression were observed between WT and RIPK3^K51A/K51A^ or MLKL^-/-^ B cells, either at baseline or following LPS stimulation (**Supplementary Figures 6C, D**).

### RIPK3 deficiency does not impair the germinal center reaction or generation of β2GPI-specific B cells following induction of a model of murine lupus

**Figure 6:**
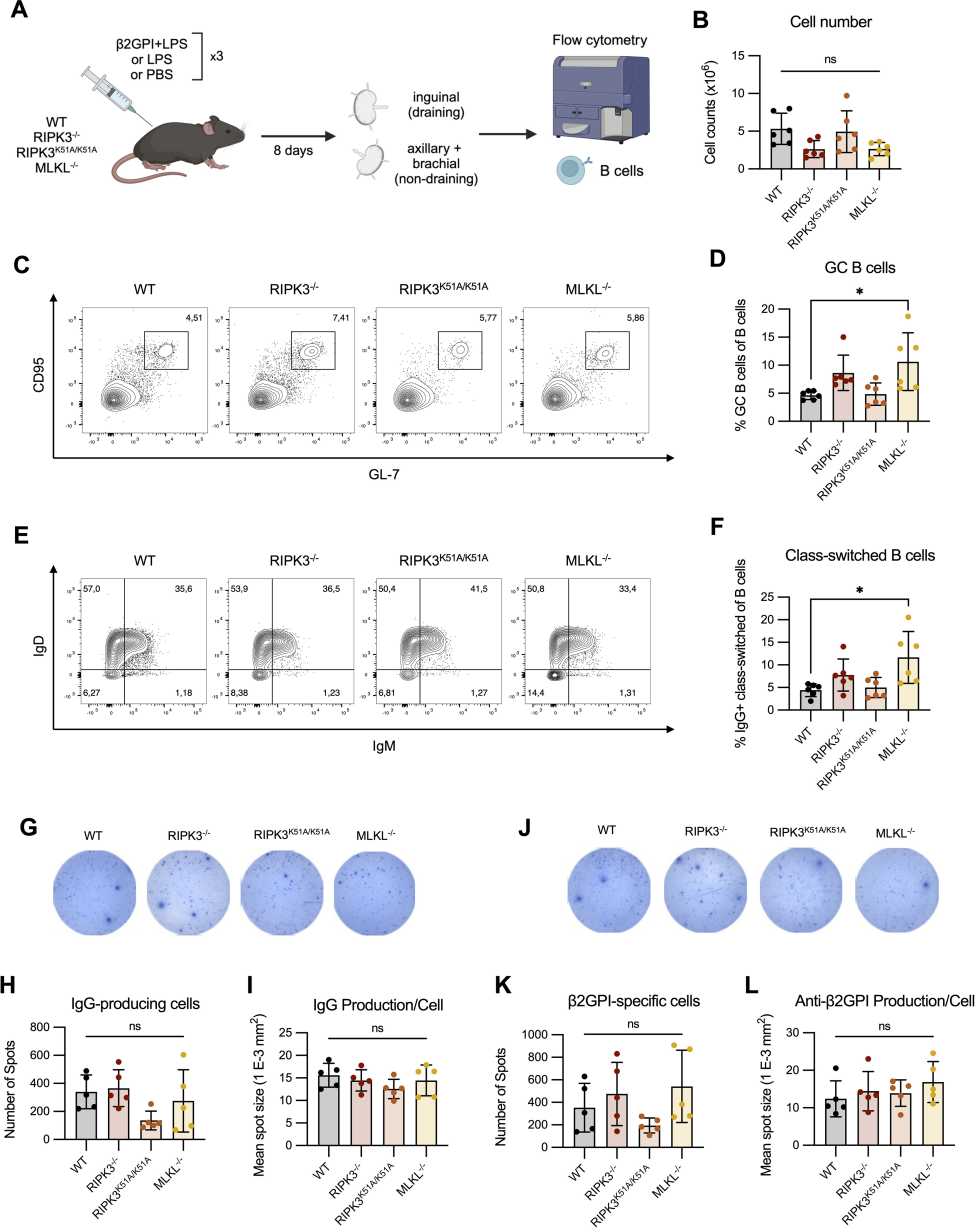
RIPK3 deficiency does not impact the generation of antigen-specific B cells following immunization with β2GPI + LPS. **(A)** WT, RIPK3^-/-^, RIPK3^K51A/K51A^ and MLKL^-/-^ mice were immunized as per induction of our model of murine lupus, for a total of 3 immunizations. Draining (inguinal) and non-draining (axillary and brachial) lymph nodes (LN) were collected 8 days following the 3^rd^ immunization. **(B-F)** Cells were counted and 1x10^6^ cells were stained for B cell markers and acquired by flow cytometry. **(B)** Cell numbers in the draining LN. **(C)** Proportion of GC (GL-7^+^ CD95^+^) B cells in the draining LN. **(D)** Proportion of IgG+ class-switched (IgD^-^ IgM^-^ IgG^+^) B cells in the draining LN. Data were pooled from two independent experiments. **(G-L)** IgG-producing and β2GPI-specific IgG-producing cells in the lymph nodes suspensions were assessed by ELISpot. **(G-I)** Representative well image **(G)**, spot count **(H)**, and mean spot size **(I)** of IgG producing cells. **(J-L)** Representative well image **(J)**, spot count **(K)**, and mean spot size **(L)** for β2GPI-specific IgG-producing cells. Data collected in a single experiment. In all graphs error bars represent mean ± SD; each data point represents an individual mouse. Data were analyzed using a one-way ANOVA, followed by a Dunnett’s *post-hoc* test to compare each group to the WT control group. *P<0.05; ns, nonsignificant.

While we were unable to observe an effect of RIPK3 deficiency on antigen presentation, its effect on the proportion of T_FH_ cells and on the level of MHC II expression by B cells following innate immune stimulation raises the possibility that deficiency in RIPK3-dependent pathways may impact the T cell-dependent maturation of B cells. We therefore investigated the proportions of GC and IgG^+^ class-switched B cells in the draining LN 8 days following immunization with β2GPI and LPS (**Figure 6A**). Total cell numbers in the draining LN were comparable between WT and RIPK3-dependent pathway-deficient mice following induction of our model of murine lupus (**Figure 6B**). No differences in the proportions of GC and IgG^+^ class-switched B cells were observed between WT and RIPK3^-/-^ or RIPK3^K51A/K51A^ mice (**Figures 6C–F**). However, an increase in the proportion of GC and IgG^+^ class-switched B cells was observed in MLKL^-/-^ mice compared to WT mice (**Figures 6C–F**). For Panels C-F in **Figure 6**, absolute cell counts were performed and did not differ from cell proportions (data not shown).

Although the proportions of GC and IgG^+^ class-switched B cells were comparable between WT and RIPK3-deficient mice, RIPK3 deficiency may specifically impact the number of β2GPI-specific IgG-producing cells. To address this possibility, we quantified total IgG and β2GPI-specific IgG production *ex vivo* using ELISpot. We found no difference in the proportion of IgG-producing cells, or in the amount of IgG produced per cell, in the draining LN of RIPK3-dependent pathway-deficient mice compared to WT mice (**Figures 6G–I**). The numbers of β2GPI-specific IgG-producing cells, as well as the per-cell production of β2GPI-specific IgG, was also comparable between WT and RIPK3-dependent pathway-deficient mice (**Figures 6J–L**).

Overall, we were unable to identify any major defects in B cell maturation or β2GPI-specific IgG-producing cells in RIPK3-deficient mice. These findings suggest that the increased proportion of T_FH_ cells and increased MHC II expression on RIPK3^-/-^ B cells did not significantly impact T cell dependent B cell maturation following induction of our model of murine lupus.

## Discussion

There is increasing evidence that RIPK3 contributes to the development of multiple inflammatory and autoimmune disorders through a variety of mechanisms, including necroptotic cell death and pro-inflammatory cytokine production (19–23). Here, we investigated which RIPK3-dependent mechanisms contribute to the generation of autoAbs in a model of murine lupus induced by repeated immunizations with β2GPI and LPS. We first established that RIPK3 deficiency dampened autoAb production in our model through mechanisms other than necroptosis and immune cell composition. Using bone marrow chimeric experiments, we then found that RIPK3 deficiency in the hematopoietic compartment resulted in lower levels of antibodies to the immunizing antigen β2GPI, but did not affect levels of autoAbs to hallmark SLE antigens. This warranted a deeper exploration into the role of RIPK3 in immune-mediated mechanisms, specifically antigen presentation.

A successful antigen-specific adaptive immune response requires a complex combination of signals and the concerted interaction of multiple immune cell types (32, 34). The responding T cell must receive three signals from the APC: [1] cell surface-expressed MHC II loaded with the specific antigenic peptide, [2] cell surface-expressed co-stimulatory molecules, and [3] secreted cytokines to direct the phenotype of the activated T cell (32, 34, 35). We were unable to detect any defects in the *in vitro* activation of T cells by RIPK3-deficient APCs. RIPK3 deficiency did not impact MHC II or co-stimulatory molecule expression by LPS-stimulated BMDCs. In addition, proliferation and IFN-γ production by antigen-specific T cells were unaffected by RIPK3 deficiency in the APC. Lastly, RIPK3 deficiency in T cells themselves did not impact their capacity to be activated *in vitro*. Together, these results suggest that RIPK3 deficiency in either APCs or T cells does not impact the outcome of antigen presentation.

In addition to signaling that is intrinsic to the immune cells involved in antigen presentation, extrinsic factors may also influence the quality of antigen presentation and subsequent T cell response (32, 34). Both the availability of antigen and the presence of external inflammatory triggers may impact the outcome of antigen presentation (36, 37). Specifically, RIPK3-dependent necroptosis may provide not only a source of SLE-specific antigens through the release of intracellular contents, but also an inflammatory trigger, as many intracellular molecules can act as danger-associated molecular patterns (DAMPs) and enhance the activation of APCs (9, 11, 12, 15). Despite our previous findings that exposure of APCs to necroptotic cells *in vitro* enhances their expression of co-stimulatory molecules and their capacity to activate β2GPI-specific T cells in an antigen-specific manner (25), we did not find an impact of RIPK3-dependent necroptosis *in vivo* in our model of murine lupus. Consistent with our previous findings (25), we found that MLKL^-/-^ and WT mice had comparable levels of autoAbs following multiple immunizations with β2GPI and LPS. It is possible that LPS obviates the need for necroptotic cell exposure as it is a stronger innate immune activator than DAMPs released from necroptotic cells. Overall, our results suggest that necroptotic cell exposure in the context of antigen presentation is not a major contributor to autoAb generation in our model of murine lupus.

In order to investigate the role of RIPK3-deficiency on antigen presentation in a more physiological context, in which extrinsic factors other than necroptosis may modulate the outcome of antigen presentation, we characterized the *ex vivo* T cell response following induction of our model. Once again, the T cell response was comparable between RIPK3-deficient and WT mice. Thus, the contribution of RIPK3 to autoAb generation in our induced model of murine lupus appears to be independent of effects on antigen presentation.

Despite the absence of a role for necroptosis in the context of our induced model of murine lupus, we noted several differences between MLKL^-/-^ and WT mice throughout our experiments. These differences were statistically significant despite the variablility we observed in MLKL^-/-^ mice. Importantly, these differences were never observed in conjunction with RIPK3-dependent effects. Increasing evidence suggests that the role of MLKL extends beyond the induction of necroptosis, and that its RIPK3-independent effects include receptor internalization and leukocyte adhesion (38–40). We previously reported that induction of murine lupus in MLKL^-/-^ mice resulted in lower levels of autoAb following the 1^st^ immunization with β2GPI and LPS (25). As described (25) and replicated here, MLKL deficiency does not impair autoAb generation following complete induction of murine lupus in our model. This finding suggests that the early impairment of autoAb generation in MLKL^-/-^ mice may be a consequence of RIPK3-independent roles of MLKL (38–40). Together, these findings suggest that RIPK3-dependent MLKL signaling is dispensable for autoAb generation following the complete induction of our model, and that RIPK3 deficiency impairs autoAb generation through mechanisms other than necroptosis.

While our results suggest that RIPK3-dependent autoAb production does not depend on antigen presentation or necroptosis, we explored RIPK3-dependent mechanisms that may impact autoAb generation. A role for RIPK3-dependent kinase activity in pro-inflammatory cytokine production by BMDCs following LPS stimulation has been described previously (41, 42). Similarly, RIPK3 deficiency has been associated with decreased serum levels of pro-inflammatory cytokines, including IL-6 and TNF-α (25, 42), as well as IFN-I (18, 25). Previously (25), we showed that RIPK3^-/-^ and MLKL^-/-^ mice produced significantly lower levels of IFN-β, IL-6, and TNF-α following a single injection of LPS, as compared to WT mice. While in the present study we did not observe any significant differences among strains in the production of IL-6 and TNF-α by BMDCs, pro-inflammatory cytokine and IFN-I production still represent a RIPK3-dependent mechanism that may contribute to the generation of autoAb in our model of murine lupus.

Our bone marrow chimera experiments imply that the presence of RIPK3 in non-hematopoietic cells is important for epitope spread in our induced model of murine lupus, since RIPK3 deficiency in the hematopoietic compartment did not significantly impair the generation of autoAbs to hallmark SLE autoantigens. These findings are consistent with those observed in other induced models of inflammatory injury (20, 42). Bone marrow chimera experiments using RIPK3^-/-^ and WT mice revealed a partial contribution of the non-hematopoietic compartment to dextran sodium sulfate-induced colitis (42). Moreover, a crucial role for necroptosis-independent RIPK3 signaling in keratinocyte activation and inflammation was identified in a murine model of psoriatic dermatitis (20). One potential mechanism by which non-hematopoietic cells may contribute to autoAb generation in our model is through RIPK3-dependent production of inflammatory cytokines and IFN-I, since these cytokines have the potential to influence autoAb generation and their expression is not exclusive to the hematopoietic compartment (43–47). Experiments in which the hematopoietic cells of RIPK3^-/-^ mice are replaced by either WT or RIPK3^-/-^ hematopoietic cells, as well as the approach we used (WT hematopoietic cells replaced by RIPK3^-/-^ or WT cells), will be critical to study this question. Our current findings are limited by the lack of a bone marrow chimera in which the role of RIPK3^-/-^ cells in both the hematopoietic and non-hematopoietic compartments is evaluated.

A limitation to our findings is that each deficient strain was compared to C57BL/6 WT mice (the background strain) rather than to its littermate control. However, we demonstrated that the WT littermate controls of RIPK3^K51A/K51A^ mice were comparable to C57BL/6 WT mice in their production of autoAbs following induction of our model of murine lupus. We acknowledge that existing genetic background variation between strains may impact the phenotype of the mutant strain. Using C57BL/6 WT mice as a common control allowed for a clear comparison with all deficient strains, rather than multiple individual comparisons between each mutant strain and its littermate control.

In conclusion, we have found that RIPK3 impacts the development of autoAbs in an induced model of murine lupus through mechanisms that are independent of antigen presentation and necroptosis. The effects of RIPK3 appear to be mediated through both hematopoietic and non-hematopoietic cells. An understanding of the specific RIPK3-dependent pathways that underlie autoAb generation and epitope spread may reveal drug-targetable pathways for the treatment and prevention of SLE and other autoimmune conditions.

## Data Availability

The raw data supporting the conclusions of this article will be made available by the authors, without undue reservation.

## Supplementary material

Supplementary Figure 1 C57BL/6 WT mice and RIPK3^K51A/K51A^ WT littermates have similar levels of autoAbs following immunization with β2GPI+LPS. C57BL/6 WT and RIPK3^K51A/K51A^ WT littermate (RIPK3^WT/WT^) mice were immunized subcutaneously with β2GPI (20ug) and LPS (10μg) for a total of 2 immunizations. Antibodies to β2GPI, CL, and DNA were measured by ELISA. In all graphs, error bars represent mean ± SD; each data point represents an individual mouse. The data shown are from one experiment. Data were analyzed using a one-way ANOVA, followed by a Tukey *post-hoc* test to compare C57BL/6 WT with RIPK3^K51A/K51A^ WT littermates. ns=not significant.

Supplementary Figure 2 RIPK3-deficient mice have an immune cell composition comparable to WT at baseline. Flow cytometry was performed on cell suspensions obtained from the **(A)** lymph nodes (pooled axillary, brachial and inguinal) and **(B)** spleen of WT, RIPK3^-/-^, RIPK3^K51A/K51A^ and MLKL^-/-^ mice at baseline. Cell numbers, and proportions of conventional (CD11c^hi^) dendritic cells, T cells, and B cells were quantified. Data were pooled from 3 independent experiments. In all graphs error bars represent mean ± SD; each data point represents an individual mouse. Data were analyzed using a one-way ANOVA, followed by a Dunnett’s *post-hoc* test to compare each group to the WT control group. ns=nonsignificant.

Supplementary Figure 3 Immunization with β2GPI+LPS results in an expansion of T_FH_ CD4^+^ cells and increased expression of MHC II on B cells. WT mice were immunized with LPS or β2GPI+LPS as per induction of our model of murine lupus, for a total of 4 immunizations. Draining (inguinal) and non-draining (axillary and brachial) lymph nodes (LN) were collected 2 days following the 4^th^ immunization. Cell suspensions were counted and 1x10^6^ cells were stained for T and B cell markers and acquired by flow cytometry. **(A)** Proportion of activated (MHC II ^hi^ CD86^hi^) DCs in the draining LN. **(B)** Proportion of IFN-γ-producing CD4^+^ T cells in the draining LN. **(C, D)** Proportion of T_FH_ (CXCR5^+^ PD-1^+^) CD4^+^ T cells in the draining and non-draining LN. **(E, F)** Level of expression of MHC II on B cells in the draining **(E)** and non-draining **(F)** LN. Data generated in a single experiment. In all graphs error bars represent mean ± SD; each data point represents an individual mouse. Data were analyzed using an unpaired t-test. *P<0.05 and ****P<0.0001; ns=nonsignificant.

Supplementary Figure 4 Immunization with β2GPI+LPS promotes B cell maturation through the germinal center reaction. WT mice were immunized subcutaneously with β2GPI (20μg) and LPS (10μg), LPS only (10µg), or PBS as per induction of our model of murine lupus, for a total of 3 immunizations. Draining (inguinal) and non-draining (axillary and brachial) lymph nodes (LNs) were collected 8 days following the 3^rd^ immunization. Cells suspensions were counted and 1x10^6^ cells were stained for B cell markers and acquired by flow cytometry. **(A)** Cell numbers in the draining LN. **(B)** Proportion of GC (GL-7^+^ CD95^+^) B cells in the draining LN. **(C)** Proportion of IgG^+^ class-switched (IgD^-^ IgM^-^ IgG^+^) B cells in the draining LN. Data were pooled from two independent experiments. In all graphs error bars represent mean ± SD; each data point represents an individual mouse. Data was analyzed using a one-way ANOVA, followed by a Dunnett’s *post-hoc* test to compare each group to the β2GPI+LPS-immunized group. *P<0.05, **P<0.01 and ****P<0.0001; and ns=nonsignificant.

Supplementary Figure 5 RIPK3 deficiency does not impact T cell proliferation or cytokine production following activation. Splenic T cells were isolated from WT, RIPK3^-/-^, RIPK3^K51A/K51A^ and MLKL^-/-^ mice, stained with CTV and stimulated with anti-CD3 and anti-CD28 for 72h. **(A, B)** Division and proliferation index of splenic CD4^+^ **(A)** and CD8^+^ **(B)** T cells were assayed by flow cytometry. **(C)** IFN-γ production in the culture supernatant was assayed by ELISA. Data were pooled from 3 independent experiments. In all graphs error bars represent mean ± SD; each data point represents an independent experiment. Data were analyzed using a one-way ANOVA, followed by a Dunnett’s *post-hoc* test to compare each group to the WT control group. ns=nonsignificant

Supplementary Figure 6 RIPK3-deficient B cells have an increased expression of MHC II following LPS stimulation. Splenic B cells were isolated from WT, RIPK3^-/-^, RIPK3^K51A/K51A^ and MLKL^-/-^ mice and cultured for 24h in the absence **(A, B)** or presence **(C, D)** of LPS (10 ng/mL). Surface expression of MHC-II, CD80 and CD86 was quantified by flow cytometry. **(A, B)** Representative histograms **(A)**, and mean fluorescence intensity (MFI) **(B)** of MHC-II, CD80 and CD86 expression on unstimulated B cells. Data were pooled from 4 independent experiments. **(C, D)** Representative histograms **(C)**, and mean fluorescence intensity (MFI) (D) of MHC-II, CD80 and CD86 expression on LPS-stimulated B cells. Data were pooled from 3 independent experiments. In all graphs error bars represent mean ± SD; each data point represents an independent experiment. Data was analyzed using a one-way ANOVA, followed by a Dunnett’s *post-hoc* test to compare each group to the WT control group. *P<0.05; ns=nonsignificant.

Supplementary Table 1 Immune cell proportions in WT and RIPK3-dependent pathways deficient mice. Immune cell proportions were quantified in the spleen, lymph nodes (LN; pooled axillary, brachial and inguinal) and thymus of WT, RIPK3^-/-^, RIPK3^K51A/K51A^, and MLKL^-/-^ mice was performed. This table summarizes the data pooled from three independent experiments. The cell type and subsets are expressed as a proportion of live cells. The mean expression levels of CD5 were assessed on TCRβ^+^ cells in the spleen and LN, and non-B cell thymocytes in the thymus. Data was analyzed by one-way ANOVA. ns=nonsignificant, nd=not determined, MFI (mean fluorescence intensity).
