# Supplementary figures and images for: RIPK3 impacts antibody generation in an induced model of murine lupus through mechanisms other than necroptosis and antigen presentation
Source: Front Immunol. 2025 Sep 30;16:1506124. doi: 10.3389/fimmu.2025.1506124 (PMC12518380; doi:10.3389/fimmu.2025.1506124)

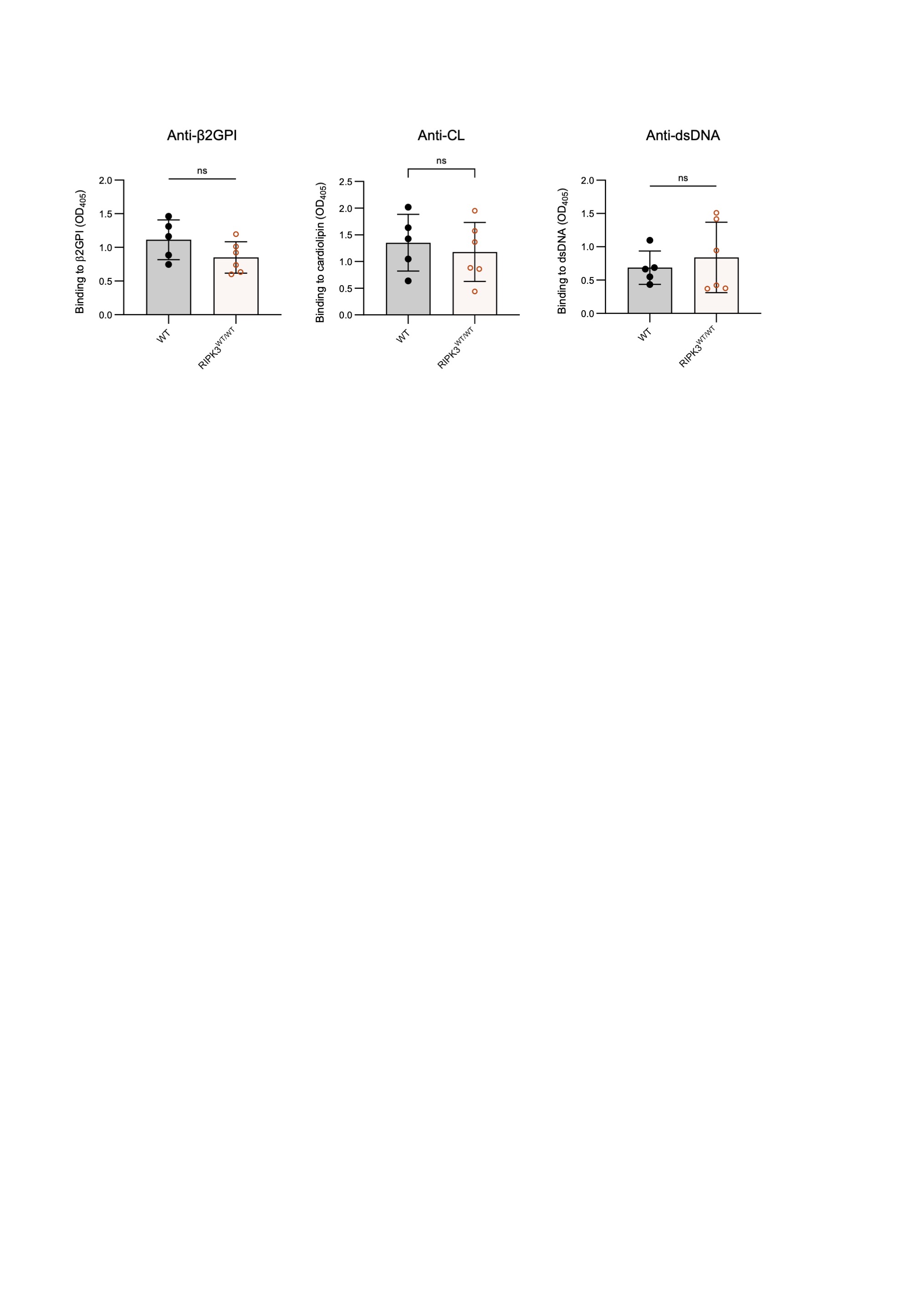

Supplement: Supplementary Figure 1 — C57BL/6 WT mice and RIPK3K51A/K51A WT littermates have similar levels of autoAbs following immunization with β2GPI+LPS. C57BL/6 WT and RIPK3K51A/K51A WT littermate (RIPK3WT/WT) mice were immunized subcutaneously with β2GPI (20ug) and LPS (10μg) for a total of 2 immunizations. Antibodies to β2GPI, CL, and DNA were measured by ELISA. In all graphs, error bars represent mean ± SD; each data point represents an individual mouse. The data shown are from one experiment. Data were analyzed using a one-way ANOVA, followed by a Tukey post-hoc test to compare C57BL/6 WT with RIPK3K51A/K51A WT littermates. ns=not significant. [file Image1.jpeg]
